# Supplementary material for: Acute effect of low-load resistance exercise with blood flow restriction on oxidative stress biomarkers: A systematic review and meta-analysis
Source: PLoS One. 2023 Apr 21;18(4):e0283237. doi: 10.1371/journal.pone.0283237 (PMC10121002; doi:10.1371/journal.pone.0283237)
Supplement: S1 Table — GRADE: Grades of Recommendation, Assessment, Development and Evaluation; SMD: Standardized mean difference; MD: Mean difference; BFR: Blood flow restriction HLRE: High-load resistance exercise LLRE: Low-load resistance exercise GSSG: Oxidized glutathione GSH: Reduced glutathione*More than 25% of participants from studies with low methodological quality (Physiotherapy Evidence Database score < 6 points).# Whether more 50% of participants were not similar to those about whom conclusions are drawn (I2 > 50%)† 75% of participants or less from studies with findings in the same direction.‡ Fewer than 400 participants for each outcome. n/a: Not applicable; was not performed due to the insufficient number of studies (<10 studies). (DOCX) [file pone.0283237.s002.docx]

**Table S1**. Certainty of evidence (GRADE system)

| **Pooled estimate**  **Damage to lipids**  **BFR versus HLT** |  |  | | **Certainty assessment** | | | | **Participants (n)** | | **Effect** | **Certainty** |
| --- | --- | --- | --- | --- | --- | --- | --- | --- | --- | --- | --- |
|  | Studies | Risk of bias^*^ | Indirectness^#^ | | Inconsistent^†^ | Imprecision‡ | Reporting Bias§ | BFR  Exercise | Conventional  Training | Difference  [95% IC] |  |
| **Post Exercise** | 3 RCTs | Limitation | No limitation | | No limitation | Limitation | n/a | 30 | 29 | SMD -0.95  [-1.49, -0.40] | ⊕⊕⊝⊝  Low |
| **Post 24 hours** | 1 RCT | Limitation | No limitation | | Limitation | Limitation | n/a | 11 | 10 | MD -1.68  [-2.90, -0.46] | ⊕⊝⊝⊝  Very Low |
| **Post 48 hours** | 1 RCT | Limitation | No limitation | | Limitation | Limitation | n/a | 11 | 10 | MD -1.52  [-3.08, 0.04] | ⊕⊕⊕⊝  Very Low |
| **LLRE-BFR versus LLRE** | | | | | | | | | | | |
| **Post Exercise** | 3 RCTs | Limitation | No limitation | | No limitation | Limitation | n/a | 29 | 26 | SMD -0.48  [-1.34, 0.38] | ⊕⊕⊝⊝  Low |
| **Post 24 hours** | 3 RCTs | Limitation | No limitation | | No limitation | Limitation | n/a | 29 | 26 | SMD -0.07  [-0.60, 0.46] | ⊕⊕⊝⊝  Low |
| **Post 48 hours** | 2 RCTs | Limitation | No limitation | | No limitation | Limitation | n/a | 23 | 20 | SMD 0.24  [-0.44, 0.92] | ⊕⊕⊝⊝  Low |
| **Damage to proteins – LLRE-BFR versus HLRE** | | | | | | | | | | | |
| **Post Exercise** | 5 RCTs | Limitation | No limitation | | Limitation | Limitation | n/a | 44 | 43 | SMD -1.39  [-2.11, -0.68] | ⊕⊝⊝⊝  Very Low |
| **Post 24 hours** | 1 RCT | Limitation | No limitation | | Limitation | Limitation | n/a | 11 | 10 | MD 0.93  [-24.24, 26.10] | ⊕⊝⊝⊝  Very Low |
| **Post 48 hours** | 1 RCT | Limitation | No limitation | | No Limitation | Limitation | n/a | 11 | 10 | MD 1.48  [-30.01, 32.97] | ⊕⊝⊝⊝  Very Low |
| **LLRE-BFR versus LLRE** | | | | | | | | | | | |
| **Post Exercise** | 3 RCTs | Limitation | No limitation | | No limitation | Limitation | n/a | 29 | 26 | SMD -0.43  [-0.97, 0.11] | ⊕⊕⊝⊝  Low |
| **Post 24 hours** | 1 RCT | Limitation | No limitation | | No limitation | Limitation | n/a | 11 | 8 | MD -1.70  [-30.31, 26.91] | ⊕⊝⊝⊝  Very Low |
| **Post 48 hours** | 1 RCT | Limitation | No limitation | | No limitation | Limitation | n/a | 11 | 8 | MD 22.57  [-9.99, 55.13] | ⊕⊝⊝⊝  Very Low |
| **Xanthine Oxidase Activity – LLRE-BFR versus HLRE** | | | | | | | | | | | |
| **Post Exercise** | 1 RCT | Limitation | No limitation | | Limitation | Limitation | n/a | 12 | 12 | MD 0.5  [-9.59, 10.59] | ⊕⊝⊝⊝  Very Low |
| **LLRE-BFR versus LLRE** | | | | | | | | | | | |
| **Post Exercise** | 1 RCT | Limitation | No limitation | | Limitation | Limitation | n/a | 12 | 12 | MD 1.13  [-11.05, 8.79] | ⊕⊝⊝⊝  Very Low |
| **Total ROS – LLRE-BFR versus HLRE** | | | | | | | | | | | |
| **Post Exercise** | 1 RCT | Limitation | No limitation | | Limitation | Limitation | n/a | 15 | 15 | MD – 0.08  [-0.17, 0.01] | ⊕⊝⊝⊝  Very Low |
| **LLRE-BFR versus LLRE** | | | | | | | | | | | |
| **Post Exercise** | 1 RCT | Limitation | No limitation | | Limitation | Limitation | n/a | 15 | 15 | MD 0.006  [-0.09, 0.11] | ⊕⊝⊝⊝  Very Low |
| **Nitric Oxide – LLRE-BFR versus HLRE** | | | | | | | | | | | |
| **Post Exercise** | 3 RCT | Limitation | No limitation | | Limitation | Limitation | n/a | 33 | 31 | SMD 0.31  [-0.20, 0.82] | ⊕⊕⊝⊝  Low |
| **LLRE-BFR versus LLRE** | | | | | | | | | | | |
| **Post Exercise** | 1 RCT | Limitation | No limitation | | Limitation | Limitation | n/a | 11 | 11 | MD 2.23  [-10.85, 15,31] | ⊕⊝⊝⊝  Very Low |
| **Catalase Activity – LLRE-BFR versus HLRE** | | | | | | | | | | | |
| **Post Exercise** | 2 RCT | Limitation | No limitation | | No limitation | Limitation | n/a | 22 | 21 | MD -0.25  [-1.81, 1.30] | ⊕⊕⊝⊝  Low |
| **Post 24 hours** | 1 RCT | Limitation | No limitation | | Limitation | Limitation | n/a | 11 | 10 | MD -2.41  [-4.55, -0.26] | ⊕⊝⊝⊝  Very Low |
| **Post 48 hours** | 1 RCT | Limitation | No limitation | | Limitation | Limitation | n/a | 11 | 10 | MD 0.03  [-2.18, 2.24] | ⊕⊝⊝⊝  Very Low |
| **LLRE-BFR versus LLRE** | | | | | | | | | | | |
| **Post Exercise** | 2 RCT | Limitation | No limitation | | Limitation | Limitation | n/a | 28 | 25 | MD 0.15  [-2.87, 3.16] | ⊕⊝⊝⊝  Very Low |
| **Post 24 hours** | 1 RCT | Limitation | No limitation | | Limitation | Limitation | n/a | 11 | 8 | MD -1.25  [-10.26, 7.76] | ⊕⊕⊝⊝  Very Low |
| **Post 48 hours** | 1 RCT | Limitation | No limitation | | Limitation | Limitation | n/a | 11 | 8 | MD 8.65  [4.63, 12.66] | ⊕⊕⊝⊝  Very Low |
| **SOD activity – LLRE-BFR versus HLRE** | | | | | | | | | | | |
| **Post Exercise** | 1 RCT | Limitation | No limitation | | Limitation | Limitation | n/a | 11 | 10 | MD -0.87  [-1.65, -0.08] | ⊕⊝⊝⊝  Very Low |
| **Post 24 hours** | 1 RCT | Limitation | No limitation | | Limitation | Limitation | n/a | 11 | 10 | MD -1.01  [-1.92, -0.09] | ⊕⊝⊝⊝  Very Low |
| **Post 48 hours** | 1 RCT | Limitation | No limitation | | Limitation | Limitation | n/a | 11 | 10 | MD -0.29  [-1.20, 0.62] | ⊕⊝⊝⊝  Very Low |
| **LLRE-BFR versus LLRE** | | | | | | | | | | | |
| **Post Exercise** | 1 RCT | Limitation | No limitation | | Limitation | Limitation | n/a | 11 | 8 | MD -0.41  [-0.96, 0.14] | ⊕⊝⊝⊝  Very Low |
| **Post 24 hours** | 1 RCT | Limitation | No limitation | | Limitation | Limitation | n/a | 11 | 8 | MD 0.27  [0.35, -0.89] | ⊕⊝⊝⊝  Very Low |
| **Post 48 hours** | 1 RCT | Limitation | No limitation | | Limitation | Limitation | n/a | 11 | 8 | MD -0.59  [-1.58, 0.40] | ⊕⊝⊝⊝  Very Low |
| **Sulfhydryl/Thiol – LLRE-BFR versus HLRE** | | | | | | | | | | | |
| **Post Exercise** | 2 RCT | Limitation | No limitation | | No limitation | Limitation | n/a | 20 | 19 | SMD 0.29  [-0.38, 0.96] | ⊕⊕⊝⊝  Low |
| **Post 24 hours** | 1 RCT | Limitation | No limitation | | Limitation | Limitation | n/a | 11 | 10 | MD 0.94  [-0.42, 2.30] | ⊕⊝⊝⊝  Very Low |
| **Post 48 hours** | 1 RCT | Limitation | No limitation | | Limitation | Limitation | n/a | 11 | 10 | MD 0.08  [-1.38, 1.54] | ⊕⊝⊝⊝  Very Low |
| **LLRE-BFR versus LLRE** | | | | | | | | | | | |
| **Post Exercise** | 2 RCT | Limitation | No limitation | | No limitation | Limitation | n/a | 17 | 14 | MD 0.27  [-0.48, 1.02] | ⊕⊕⊝⊝  Low |
| **Post 24 hours** | 1 RCT | Limitation | No limitation | | Limitation | Limitation | n/a | 11 | 8 | MD 0.45  [-1.46, 2.36] | ⊕⊝⊝⊝  Very Low |
| **Post 48 hours** | 1 RCT | Limitation | No limitation | | Limitation | Limitation | n/a | 11 | 8 | MD 0.00  [-1.27, 1.27] | ⊕⊝⊝⊝  Very Low |
| **Total Glutathione- LLRE-BFR versus HLRE** | | | | | | | | | | | |
| **Post Exercise** | 1 RCT | Limitation | No limitation | | No limitation | Limitation | n/a | 10 | 10 | See Table 3 | ⊕⊕⊝⊝  Low |
| **Post 24 hours** | 1 RCT | Limitation | No limitation | | Limitation | Limitation | n/a | 10 | 10 | See Table 3 | ⊕⊝⊝⊝  Very Low |
| **Uric Acid – LLRE-BFR versus HLRE** | | | | | | | | | | | |
| **Post Exercise** | 1 RCT | Limitation | No limitation | | No limitation | Limitation | n/a | 10 | 10 | MD 0.44  [-2.54, 3.42] | ⊕⊝⊝⊝  Very Low |
| **LLRE-BFR versus LLRE** | | | | | | | | | | | |
| **Post Exercise** | 1 RCT | Limitation | No limitation | | No limitation | Limitation | n/a | 6 | 6 | MD -0.12  [-2.18, 1,94] | ⊕⊝⊝⊝  Very Low |
| **Total Antioxidant Capacity – LLRE-BFR versus HLRE** | | | | | | | | | | | |
| **Post Exercise** | 3 RCT | No limitation | No limitation | | Limitation | Limitation | n/a | 29 | 29 | Not pooled,  See Table 3 | ⊕⊕⊝⊝  Low |
| **Post 24 hours** | 1 RCT | Limitation | No limitation | | No limitation | Limitation | n/a | 10 | 10 | See Table 3 | ⊕⊝⊝⊝  Low |
| **LLRE-BFR versus LLRE** | | | | | | | | | | | |
| **Post Exercise** | 1 RCT | Limitation | No limitation | | Limitation | Limitation | n/a | 24 | 24 | MD 0.50  [-1.21, 2.21] | ⊕⊝⊝⊝  Very Low |
| **Post 24 hours** | 1 RCT | No limitation | No limitation | | Limitation | Limitation | n/a | 12 | 12 | MD -7.15  [-19.54, 5.24] | ⊕⊕⊝⊝  Low |
| **Post 48 hours** | 1 RCT | No limitation | No limitation | | Limitation | Limitation | n/a | 12 | 12 | MD -3.51  [-19.98, 12,96] | ⊕⊕⊝⊝  Low |
| **GSSG value – BFR versus HLRE** | | | | | | | | | | | |
| **Post Exercise** | 1 RCT | No limitation | No limitation | | Limitation | Limitation | n/a | 9 | 9 | MD -1.66  [-3.13, 0.11] | ⊕⊕⊝⊝  Low |
| **GSH value – BFR versus HLRE** | | | | | | | | | | | |
| **Post Exercise** | 1 RCT | No limitation | No limitation | | Limitation | Limitation | n/a | 9 | 9 | MD -0.81  [-2.73, 1.11] | ⊕⊕⊝⊝  Low |
| **GSH ratio – LLRE-BFR versus HLRE** | | | | | | | | | | | |
| **Post Exercise** | 3 RCT | Limitation | No limitation | | No limitation | Limitation | n/a | 19 | 19 | SMD -1.12  [-1.70, -0.55] | ⊕⊕⊝⊝  Low |
| **LLRE-BFR versus LLRE** | | | | | | | | | | | |
| **Post Exercise** | 1 RCT | Limitation | No limitation | | Limitation | Limitation | n/a | 12 | 12 | MD 0.31  [-1.01, 1.63] | ⊕⊝⊝⊝  Very Low |

GRADE: Grades of Recommendation, Assessment, Development and Evaluation;

SMD: Standardized mean difference;

MD: Mean difference;

BFR: Blood flow restriction

HLRE: High-load resistance exercise

LLRE: Low-load resistance exercise

GSSG: Oxidized glutathione

GSH: Reduced glutathione

*More than 25% of participants from studies with low methodological quality (Physiotherapy Evidence Database score < 6 points).

# Whether more 50% of participants were not similar to those about whom conclusions are drawn (I^2^ > 50%)

† 75% of participants or less from studies with findings in the same direction.

‡ Fewer than 400 participants for each outcome.

n/a: not applicable; was not performed due to the insufficient number of studies (<10 studies).
