# Supplementary material for: Acute effect of low-load resistance exercise with blood flow restriction on oxidative stress biomarkers: A systematic review and meta-analysis
Source: PLoS One. 2023 Apr 21;18(4):e0283237. doi: 10.1371/journal.pone.0283237 (PMC10121002; doi:10.1371/journal.pone.0283237)
Supplement: S1 Search — (DOCX) [file pone.0283237.s003.docx]

Supplementary 1. Search strategy

PubMed

#1 Blood flow restriction

#2 Vascular occlusion

#3 kaatsu

#4 ischemic training

#5 occlusion training

#6 #1 OR #2 OR #3 OR #4 OR #5

#7 Oxidative stress

#8 Oxidative Stresses

#9 Anti-oxidative Stress

#10 Oxidative Damage

#11 Oxidative Stress Injury

# 12 #7 OR #8 OR #9 OR #10 OR #11

#13 Exercise

#14 Physical exercise

#15 Acute Exercise

#16 Isometric exercise

#17 Aerobic exercise

#18 Exercise Training

#19 #13 OR #14 OR #15 OR #16 OR #17 OR #18

#20 Randomized controlled trial

#21 Controlled clinical trial

#22 Comparative study

#23 Random

#24 Crossover Design

#25 Cross Over Trials

#26 Crossover Studies

#27 Placebo

#28 Trial.

#29 #20 OR #21 OR #22 OR #23 OR #24 OR #25 OR #26 OR #27 OR #28

#30 #6 AND #12 AND #19 AND #29

PeDro (Advance Search)

Abstract & Title: Oxidative stress OR Antioxidant

AND

Method: Clinical Trial

AND

Title only: Blood flow restriction OR Vascular occlusion OR Kaatsu OR Partial occlusion

CENTRAL

#1 Blood flow restriction

#2 Vascular occlusion

#3 kaatsu

#4 ischemic training

#5 occlusion training

#6 #1 OR #2 OR #3 OR #4 OR #5

#7 Oxidative stress

#8 Anti-oxidative Stress

#9 Oxidative Damage

#10 Oxidative Stress Injury

#11 #7 OR #8 OR #9 OR #10

#12 Exercise

#13 Acute Exercise

#14 Isometric exercise

#15 Aerobic exercise

#16 #12 OR #13 OR #14 OR #15

#17 #6 AND #11 AND #16

Box “Trials”

EMBASE (Esbsco)

#1 'blood flow restriction' OR (vascular AND occlusion) OR kaatsu OR (ischemic AND training)

#2 'oxidative stress' OR antioxidant OR 'oxidative damage' OR 'antioxidant activity'

#3 exercise OR 'resistance training' OR 'acute exercise' OR 'aerobic exercise' OR 'isometric exercise'

#4 'crossover procedure' OR 'randomized controlled trial' OR 'clinical trial'

#5 #1 AND #2 AND #3 AND #4

CINAHL / SPORTDiscus (Ebsco)

S1 blood flow restriction OR occlusion training OR restriction of blood flow OR partial occlusion OR kaatsu OR vascular occlusion [all text]

S2 oxidative stress OR oxidative damage OR antioxidants [all text]

S3 exercise OR physical activity OR resistance training OR aerobic exercise [all text]

S4 S1 AND S2 AND S3

Virtual Health Library (Advance Search)

(blood flow restriction OR occlusion training OR restriction of blood flow OR partial occlusion OR kaatsu OR vascular occlusion ) AND ('oxidative stress' OR antioxidant OR 'oxidative damage' OR 'antioxidant activity') AND (exercise OR 'resistance training' OR 'acute exercise' OR 'aerobic exercise' OR 'isometric exercise')
