# Supplementary material for: Acute effect of low-load resistance exercise with blood flow restriction on oxidative stress biomarkers: A systematic review and meta-analysis
Source: PLoS One. 2023 Apr 21;18(4):e0283237. doi: 10.1371/journal.pone.0283237 (PMC10121002; doi:10.1371/journal.pone.0283237)
Supplement: Attachment — Submitted filename: PONE-D-22-31577v1.doc [file pone.0283237.s004.doc]

**Model for Manuscript Review**

**Research articles**

**Manuscript title:**

PONE-D-22-31577 - Acute Effect of Low Load Resistance Exercise with Blood Flow Restriction on Oxidative Stress Biomarkers: A Systematic Review and Meta-Analysis

| **Títle** |  |
| --- | --- |
| Is it understandable and concise? | ( x ) Yes ( ) Not |
| Reflects the content? | ( x ) Yes ( ) Not |
| **Abstract** |  |
| It includes: objectives, methodology, key findings and conclusions? | ( x ) Yes ( ) Not |
| **Introduccion** |  |
| The investigation was carried out in a suitable theoretical structure? | ( x ) Yes ( ) Not |
| Clear leaves the questions you want to answer and objectives of the work? | ( x ) Yes ( ) Not |
| The cited references are current and relevant? | ( x ) Yes ( ) Not |
| **Methods** |  |
| The methods presented are appropriate to achieve the proposed objectives? | ( x ) Yes ( ) Not |
| The selection and composition of the sample are adequately described? | ( x ) Yes ( ) Not |
| The data collection process and the tools used are described clearly? | ( x ) Yes ( ) Not |
| The statistical analysis and the research design appropriate? | ( x ) Yes ( ) Not |
| **Results** |  |
| The presentation of the results clear? | ( x ) Yes ( ) Not |
| The main results are highlighted without the inclusion of interpretation and comparisons? | ( x ) Yes ( ) Not |
| The results evaluate the proposed objectives? | ( x ) Yes ( ) Not |
| Tables and figures are properly numbered, labeled and explained? | ( x ) Yes ( ) Not |
| **Discussion and Conclusion** |  |
| The results are discussed based on the literature? | ( ) Yes ( x ) Not |
| Author's interpretations show the safety and soundness? | ( x ) Yes ( ) Not |
| The limitations of the work are presented? | ( x ) Yes ( ) Not |
| The conclusions of the study are presented? | ( x ) Yes ( ) Not |
| The conclusions respond to the objectives? | ( ) Yes ( x ) Not |

**General comments:**

**Title**

Are presented satisfactorily.

**Abstract**

Are presented satisfactorily.

**Introduction**

Are presented satisfactorily.

**Methods**

Are presented satisfactorily.

**Results**

Are presented satisfactorily.

**Discussion**

Are presented satisfactorily.

**Conclusion**

Are presented satisfactorily. However, I should have the practical applications of what I found.

**References**

68 references were presented, their numbering is incorrect, the formatting needs to be revised, and 30 are outdated. please review.

**Overview**

The manuscript presented addresses a relevant research topic.

It would be advisable to do a general review.

**Specific comments and suggestions:**

**Outcome evaluation**

- Accept unchanged	(	)
- Accepted with minor changes	( x )
- Accepted with major changes	(	)
- Rejected	(	)
