# Supplementary material for: Acute effect of low-load resistance exercise with blood flow restriction on oxidative stress biomarkers: A systematic review and meta-analysis
Source: PLoS One. 2023 Apr 21;18(4):e0283237. doi: 10.1371/journal.pone.0283237 (PMC10121002; doi:10.1371/journal.pone.0283237)
Supplement: Attachment — Submitted filename: ReviewerResponses.docx [file pone.0283237.s005.docx]

Thank you to both reviewers for their comments on our manuscript. We have taken the time to go through and address all comments and in the process, reshaped the manuscript significantly based on your feedback. We wish to thank both of you for the time spent and the thoughtful comments provided to help make a more cohesive document. Our responses are in red for each of the specific comments. For your convenience, when appropriate/relevant, we have copied/pasted the specific sections of the manuscript for reference and these are presented in green. We hope that these changes make the article suitable for publication and thank you again!

Reviewer #1: The manuscript was duly revised and adjustments were made by the authors, and minor adjustments are necessary for publication, this is our technical opinion. The theoretical framework is outdated and there are still inconsistencies regarding their formatting.

**General comments:**

**Conclusion**

Are presented satisfactorily. However, I should have the practical applications of what I found.

In the new revised document, the practical applications are discussed in lines 612-631 whereas our conclusion is specifically related to the observations gleaned from our analysis. In short, we comment on the fact that LLRE-BFR may be important for those with injuries or pathologies where an imbalance in redox status is likely occurring and how LLRE-BFR does not appear to induce additional imbalances in redox status, compared to HLRE and as such, could be a suitable alternative for those populations. This is particularly true given longitudinal studies have shown LLRE-BFR induces similar muscle hypertrophy and strength gains as HLRE.

The information is copy-pasted here for your reference:

“The findings of this review can help coaches and professionals working in rehabilitation. Our findings suggest that LLRE-BFR can be a potentially useful strategy in exercise prescription, since LLRE-BFR seems to attenuate ROS-induced cell damage and injury when compared to HLRE in healthy individuals. This is mainly relevant in periods of intensive training and in the rehabilitation of musculoskeletal injuries (i.e., post-surgery conditions, in those suffering from arthritis, or in elderly frail individuals), where the chemical and enzymatic antioxidant defense system may be insufficient to combat the rise of free radicals induced by exercise (70,71). This has important implications in clinical situations, since disturbances in redox homeostasis in favor of oxidants are associated with delay muscle recovery, greater delayed onset muscle soreness, fatigue, and reduction in strength, affecting the muscle performance (14,70).

In this way, the exercise-induced oxidative stress could likewise be relevant for exercise prescription. Although a HLRE session induces greater oxidative damage, it seems unlikely that this training modality amplifies oxidative stress enough to be harmful to human health given the many physiologic benefits observed with chronic training programs. In light of this, LLRE-BFR is considered a generally safe and effective resistance exercise approach that may aid in faster muscle recovery since certain thresholds are not surpassed (i.e., load, pressure, and time under BFR) reducing the propensity to accumulate ROS while still stimulating positive musculoskeletal outcomes comparable to HLRE.”

**References**

68 references were presented, their numbering is incorrect, the formatting needs to be revised, and 30 are outdated. please review.

Thank you for the comments. We have gone into the document and completely redid our references, reformatted according to the journal’s specifications and reviewed all of our citations to determine redundancy. We kept most of them because they are relevant and provide evidence support for the statements made about reactive oxygen species and do not appear to be outdated. We did include a couple of newer references that we did not cite originally when we went back to revise the manuscript. We hope that the revisions are suitable and if you believe that newer references are still required, we will make the appropriate adjustments if possible. There are now 71 references.

**Overview**

The manuscript presented addresses a relevant research topic.

It would be advisable to do a general review.

Thank you for the comments. We have taken them into consideration and have substantially revised the manuscript for language, formatting, and content/analysis. We hope you view the new version of this manuscript favorably as we feel it is considerably improved based on your feedback.

Reviewer #2: Dear Authors,

I would like to express my gratitude regarding the opportunity to review this manuscript.

It is an interesting study, congratulations. At this stage the manuscript requires considerable improvements. Below suggestions with line or page indication.

5-30 - Please review the instructions for authors and change the text format accordingly

The manuscript was carefully revised in accordance with journal standards. We hope that the changes are in accordance with your request.

34-54 - Please revise the abstract text, namely the abbreviations format and sections (methods, results, and others), considering the instructions for authors.

We have made the appropriate changes as per journal specifications. We apologize for not meeting formatting requirements on our initial submission. Thank you.

For your convenience, the abstract is presented here:

**Abstract**

**Background**

The purpose of this review was to analyze the acute effects of low-load resistance exercise with blood flow restriction (LLE-BFR) on oxidative stress markers in healthy individuals in comparison with LLE or high-load resistance exercise (HLRE) without BFR.

**Materials and Methods**

A systematic review was performed in accordance with the PRISMA (Preferred Reporting Items for Systematic Reviews and Meta-Analyses) guidelines. These searches were performed in CENTRAL, SPORTDiscus, EMBASE, PubMed, CINAHL and Virtual Health Library- VHL, which includes Lilacs, Medline and SciELO. The risk of bias and quality of evidence were assessed through the PEDro scale and GRADE system, respectively.

**Results**

Thirteen randomized clinical trials were included in this review (total n=158 subjects). Results showed lower post-exercise damage to lipids (SMD= -0.95 CI 95% -1.49 to -0. 40, I^2^=0%, p=0.0007), proteins (SMD= -1.39 CI 95% -2.11 to -0.68, I^2^=51%, p=0.0001) and redox imbalance (SMD= -0.96 CI 95% -1.65 to -0.28, I^2^=0%, p=0.006) in favor of LLRE-BFR compared to HLRE. HLRE presents higher post-exercise superoxide dismutase activity but in the other biomarkers and time points, no significant differences between conditions were observed. For LLRE-BFR and LLRE, we found no difference between the comparisons performed at any time point.

**Conclusions**

Based on the available evidence from randomized trials, providing very low or low certainty of evidence, this review demonstrates that LLRE-BFR promotes less oxidative stress when compared to HLRE but no difference in levels of oxidative damage biomarkers and endogenous antioxidants between LLRE.

Register number: PROSPERO number: CRD42020183204

36 - “high load” / 62 - “high-load” - Please carefully revise all manuscript standardizing these details.

Thank you for noticing the differences. We have standardized “high-load” as the phrase throughout the manuscript.

58 - Please review the instructions for authors, subtopics normally do not present numbers in this journal.

We have made the appropriate adjustments to adhere to the formatting of the journal.

70 and 100 - The reference format is different. Please correct in all cases throughout the manuscript.

We have standardized the format to Vancouver citations throughout the manuscript as per the journal’s specifications

Please review line 133.

Ln 133 is the search strategy figure. We are unsure of what you would like us to do. Please clarify if this comment is still valid. In the new manuscript, it occurs on Ln 260:

**Figure 1. Flow diagram of trials in the review.** HLRE – High-load Resistance Exercise; LLRE – Low Load Resistance Exercise.

149-150 - I do not think “XXX” is adequate. It is more important to understand the authors involved in the search academic background and experience.

160, 168, 169, 177, 182 - Please consider changing “XXX”.

We have made changes to the manuscript as requested and any new text is highlighted in red. We also added the researchers initials for the reviewer. They are presented in text here:

Ln 159-61: This search strategy was used by two blinded researchers (JVF and MVF) independently. Disagreements were resolved through a third researcher (TDM) to reach a consensus.
Ln 179-81: (5) biomarkers associated with oxidative stress measured in blood was extracted by each author (JVF and MVF) independently and disagreements were resolved by a third researcher (TDM).

Ln 194: The study information was extracted independently by the researchers (JVF and MVF)

Ln 230: Two reviewers (JVF and TDM) assessed the quality of the current evidence using

174-175 - Please standardize the text format “-“ or “/”.

We have made the changes to standardize it to remove ambiguity. In the revised version of the manuscript, it is on Ln 192: considered 'poor', 4 or 5 'fair', 6, 7, or 8 'good,' and 9 or 10 'excellent'. For trials evaluating

202 - Please include city and country.

We have added the country as the city (Copenhagen) was already inside the citation. We hope this meets your revision requirements and it is on Ln 200 in the revised manuscript: Copenhagen, Denmark: The Nordic Cochrane Center, The Cochrane Collaboration,

224 - Please provide a reference.

We have added a citation to support the Higgin’s inconsistency measure. It is reference 23 in text and presented on Ln 221: thresholds set as I^2^ = 25% (low), I^2^ = 50% (moderate), and I^2^ = 75% (high) (23).

243 - Please include end point.

We have made the adjustment on Ln 261 in the revised manuscript: LLRE – Low Load Resistance Exercise.

269 - “et al.” - end point missing. Same in lines 390, 429, 432, 433, 517 and other lines (please revise all manuscript).

We have added the end point as requested and made similar adjustments throughout the manuscript and are highlighted in red. We hope this meets your revision request.

298 - In the same line, numbers in different format. Please standardize throughout the manuscript.

We have made the adjustments and ensured the same formatting and can be found on line 858 in the new manuscript.

303 - Please reformulate the text.

We have made the changes according to the specifications of the journal and it is now within the body of the manuscript starting on line 318.

336, 342, 349 - Please include end points. This should be considered in all cases throughout the manuscript.

We have made the appropriate changes requested throughout the manuscript.

336, 342, 349 - Please consider text between figures and table.

We moved Figure 2 to begin at Line 332. This provides some text between all figures/tables. Thank you.

387 - Please correct “[40,41,]”.

We have made the adjustments in the revision. It is shown here on Ln 400 in the revised manuscript: catalase (38,39,43), as well as non-enzymatic antioxidants total glutathione (36) and uric

511 – “Kolind [54]” - incorrect citation format. Please correct.

We have made the adjustments in the revision. It is now cited correctly on Ln 522: antioxidant and oxidative stress responses. Another study by Kolind et al. (56) supports

556 - It seems more than one space after end point, please revise.

We actually removed this from the manuscript as it was not needed given some of the new edits.

586-604 - This and other paragraphs are too long and do not favor reading. Please consider splitting.

We split the paragraph in half to facilitate a better reading experience. Starting on Ln 596-613 it reads:

It is well established that trained individuals are less susceptible to oxidative damage than untrained individuals due to a greater antioxidant defense capacity (69). Therefore, untrained subjects are less effective at neutralizing ROS generated during exercise and may indicate that antioxidant enzymes are regulated by the redox status and not by the exercise itself, as suggested by Bessa et al. (14). However, the current literature does not allow us to draw a firm conclusion on whether training status can influence LLRE-BFR-mediated redox responses. Investigating the effects of LLRE-BFR training on oxidative stress markers in both untrained and trained populations could further elucidate the relevancy of ROS and subsequent oxidative stress to the adaptation processes following LLRE-BFR.

Furthermore, there are no studies comparing the effects of the LLRE-BFR protocol to muscle failure versus a predefined repetition protocol on oxidative stress markers, but we observed that protocols conducted to failure or non-failure with LLRE-BFR were less likely to induce oxidative stress than HLRE. In contrast, we observed a tendency in HLRE to induce oxidative stress regardless of training status (42,44). Finally, studies elucidating the effects of a long-term LLRE-BFR protocol on the modulation of redox system responses are needed and will help to understand the magnitude of LLRE-BFR in the generation of ROS.

633-635 - “secondly” and “thirdly” suggested.

We made the changes as requested and they’re presented on Ln 641-643: PEDro score of ~5.53. Secondly, we included only up to three time points (post-, 24-, 48 hours) of oxidative marker responses. Conclusions on the time-course of oxidative stress outside of this period are scarce. Thirdly, we did not include studies that used aerobic

663 - After conclusions section and before references, please consider the journal template, namely including Author Contribution.

We have added this on Ln 672-686.

666 – Please carefully revise all references format and change according to the instructions for author’s format.

All changes have been made to meet journal reference specifications.

Page 36 – Figure 1 - Some incorrections seem to be present in figure. The “identification” box, the position of “=” is not standardized and aside of “included” box it seems to appear a word track change. Please carefully revise the figure content.

We made all the changes requested in Figure 1.

38 - All the other figures should be corrected, namely including “et al.” and improving the figures quality. The figures footers (legends) should also be carefully revised.

We made all the changes requested in Figure 1 as well as in all other figures (adding et al. as well as making sure all figures are congruent. We hope the changes are sufficient. We also removed all captions in the figure section as this is not within journal guidelines.

Please carefully revise the English throughout the manuscript.

The manuscript was carefully and extensively revised, I hope that the changes are in accordance with your request.
