# Supplementary material for: Acute effect of low-load resistance exercise with blood flow restriction on oxidative stress biomarkers: A systematic review and meta-analysis
Source: PLoS One. 2023 Apr 21;18(4):e0283237. doi: 10.1371/journal.pone.0283237 (PMC10121002; doi:10.1371/journal.pone.0283237)
Supplement: Attachment — Submitted filename: ResponseToReviewers_PR2.docx [file pone.0283237.s006.docx]

**Reviewer #2**

11, 13, 17 – Please revise the text format throughout the manuscript. One example in these 3 lines, the text starts with different formats.

**Our response:** Thank you, this has been amended.

72 and 73 – Please correct the numbers and text format.

**Our response:** Thank you, this has been amended.

119 – “and” seems appropriated before “2)”. Please consider.

**Our response:** Thank you, this has been amended.

136 – It is suggested MVC in full in the first appearance in the manuscript, but afterwards, in lines 138 and 139, only the abbreviation.

**Our response:** Thank you, this has been amended.

173 – It seems a line spacing is missing, please revise.

**Our response:** Thank you, this has been amended.

256 – “(39,41,43).”, although, citation 36-42 only in line 267. Please carefully revise because this compromises all the reference and citation numbers.

**Our response:** Thank you, this has been amended to reflect accurate citation numbers.

262, 263 – It looks like more than one line spacing, please revise.

**Our response:** Thank you, this has been amended.

274-275 – I believe word track changes emerge close to line number, please revise.

**Our response:** Thank you, this has been amended.

299 – Please revise the table content. Some text alignment in central, other not. Standardization according to the journal instructions for authors is suggested. Also, the text paragraph size is not standardized, please carefully revise. Please also revise all tables format (lines and other details).

**Our response:** Thank you, this has been amended.

301 – All abbreviations should be in full in table footnote.

**Our response:** Thank you, this has been amended (line 305).

318 - Some text alignment in central, other not. Standardization according to the journal instructions for authors is suggested in table 2.

**Our response:** Thank you, this has been amended.

319 – Please revise text format.

**Our response:** Thank you, this has been amended.

355 – Table 1 footnote presents end point, table 2 not, please standardize considering the journal instructions for authors.

**Our response:** Thank you, this has been amended.

431 – “24 hours” suggested instead of “24-“.

**Our response:** Thank you, this has been amended.

479/480 – “heavy load resistance exercise (HLRE)” 460 – “High-load resistance exercise”. Please revise all manuscript considering the correction of these details.

**Our response:** Thank you, this has been amended.

660 – Please insert line spacing.

**Our response:** Thank you, this has been amended.

Please carefully revise all references format. For example, ref 1 is incorrect. “internet” and link unnecessary, also volume and number in wrong format, “30(15)” in this particular example.

**Our response:** We have removed all “[internet]” mentions in the references list. We formatted our references in accordance with “Vancouver” format as recommended by the journal’s guidelines so we are unsure

Please carefully revise the English throughout the manuscript.

**Our response:** We have a primary English speaker as a lead author and have gone through the manuscript in another round of revisions. Any changes are made in red throughout the manuscript with track changes. We feel the manuscript articulates our points effectively, but if you feel there is specific sections that are not clear after these revisions, can you please point them out so we can further scrutinize to improve our communication of these topics? Thank you.

**Reviewer #3:**

Dear authors,

All the points raised by he previous reviewers were satisfactory answered by your team. This manuscript follows the higher standards in conducting systematic reviews and addresses a topic of interest to the readers and to scientific community.

I have no further comments to add.

Best regards,

**Our response:** Our sincere thanks for your review and the positive feedback on the article.

**Reviewer #4:**

1- The reviewed oxidative stress markers are not included in the abstract

**Our response:** In this review, we observed that more than 10 different biomarkers were used to assess oxidative stress. Therefore, we chose to summarize the main findings of the review in the abstract. Thus, we followed the guidelines of PLOS ONE regarding the writing of the abstract.

The Abstract should: *" (a) Describe the main objective(s) of the study; (b) Explain how the study was done, including any model organisms used, without methodological detail; (c) Summarize the most important results and their significance; (d) Not exceed 300 words"*

2- The keywords have not been selected properly

**Our response**: Thank you, this has been amended.

“Keywords: reactive oxygen species, free radicals, ischemic training, exercise, resistance training”

3- The introduction is too long, Also, it should be mentioned what kind of oxidative stress markers are induced by exercise and what are the mechanisms of inducing them.

**Our response:** Thank you for your comment, we added a sentence to help readers better understand this topic.

*“Furthermore, after resistance exercise, physiological responses such as the infiltration of phagocytes (ie, neutrophils and macrophages) at the site of injury are necessary for neuromuscular adaptation. This exercise-induced inflammatory response also contributes to increased free radical production, contributing to oxidative damage to biomolecules.”*

However, in our introduction, we approach the relationship between oxidative stress and exercise in a summarized way, since the literature presents a diversity of tests and markers that can be used to measure the levels of oxidative stress. Furthermore, exercise-induced ROS production comes from different sources, as described in the introduction. In our view, addressing each biomarker and the mechanism involved would make the introduction extremely long, not following the journal's recommendations.

Thus, in the introduction, we chose to maintain the journal's recommendations: *"Provide a background that contextualizes the manuscript and allows readers outside the area to understand the purpose and meaning of the study, as well as a brief review of the literature."*

4- Instead of TBARS please mention MDA

**Our response:** Although both the TBARS and MDA assays are both used to estimate the lipid damage caused by the increase in reactive species induced by muscle activity during exercise, it is already well evidenced in the literature that the TBARS assay can interact with a variety of oxidized lipids, both saturated and unsaturated aldehydes, sucrose and urea to form various chromogens, thus not specifically measuring just MDA. For this reason and based on the current literature, we chose not to make changes to the table referring to oxidative stress markers.

Forman et al, 2015: *“Thus, one cannot directly equate the measurement of TBARS with MDA or lipid peroxidation when measured in a complex biological system.”*

Forman HJ, Augusto O, Brigelius-Flohe R, et al. Even free radicals should follow some rules: a guide to free radical research terminology and methodology. Free Radic Biol Med. 2015;78:233-235. doi:10.1016/j.freeradbiomed.2014.10.504

Moselhy HF, Reid RG, Yousef S, Boyle SP. A specific, accurate, and sensitive measure of total plasma malondialdehyde by HPLC. J Lipid Res. 2013;54(3):852-858. doi:10.1194/jlr.D032698

5- In addition to number of papers please add the number of subjects.

**Our response:** Thank you, this has been amended throughout the manuscript.

6- Publication bias evaluation is important and to investigate the presence of publication bias in the meta-analysis, the funnel plot, Begg’s rank correlation and Egger’s weighted regression tests can be used. Please add funnel plot analysis.

**Our response:** We partial agree with the reviewer, however as described on line 247-8" publication bias that was evaluated using a funnel plot when 10 or more studies were in the same comparison". This is in line with the Cochranne Hadbook recommendations ([**https://handbook-5-1.cochrane.org/chapter_10/10_4_3_1_recommendations_on_testing_for_funnel_plot_asymmetry.htm**](https://handbook-5-1.cochrane.org/chapter_10/10_4_3_1_recommendations_on_testing_for_funnel_plot_asymmetry.htm)). As observed in the comparisons made, there were no more than 10 clinical trials combined, so we did not perform a funnel plot, avoiding a mistaken interpretation of the results.
