# Supplementary material for: Acute effect of low-load resistance exercise with blood flow restriction on oxidative stress biomarkers: A systematic review and meta-analysis
Source: PLoS One. 2023 Apr 21;18(4):e0283237. doi: 10.1371/journal.pone.0283237 (PMC10121002; doi:10.1371/journal.pone.0283237)
Supplement: Attachment — Submitted filename: Reviewer_Responses_PR3.docx [file pone.0283237.s007.docx]

Thank you for taking the time to make this manuscript the best it can be. We feel that your comments have helped shape the manuscript for the better. We hope you feel the paper is suitable for publication.

Reviewer #2

Table 1 – It is believed to be “de Lima” and not “Lima” in ref 45. Same in table 2. Please revise.

**Authors response:** Thanks for the suggestion, we've adjusted the table as requested.

Table 1 & 2 and text manuscript. Ref 39 is not “Ramis”, should be corrected in tables and manuscript text or in references. This is very important because can determine errors throughout the document.

**Authors response:** Thanks for the suggestion, we've adjusted the document as requested.

562 – Please consider not starting the phrase with the authors citation.

**Authors response:** Thanks for the suggestion, we've adjusted the sentence. It now reads:

“For example, there is evidence (62) demonstrating that low doses and short-term ROS exposure stimulate mTORC1 while high concentrations or long-term exposure ROS inhibit mTORC1 activity.”

694-987 – Please carefully analyze and correct the references format considering the journal guidelines.

**Authors response:** We have adjusted the manuscript to remove the “[Internet]” that was present that may have been the issue the reviewer pointed out. Otherwise, the manuscript is in Vancouver style according to our reference manager, Paperpile. According to PLOS: *PLOS uses the reference style outlined by the International Committee of Medical Journal Editors (ICMJE), also referred to as the “Vancouver” style.*

Please consider improving all figures quality.

**Authors response:** We are unsure what you are requesting as there is no direction provided by the reviewer. The images provided are from a computer program that is used to produce images for these types of analyses. We have already adjusted the figures in PR1 that met the satisfaction of the other reviewers (we redefined terms and standardized nomenclature used within these figures). If this is not adequate to your expectations, please specify what exactly you are requesting so we can make the changes.

Please revise the manuscript considering English details improvment.

**Authors response:** We have gone through and adjusted the manuscript once more and the new edits are in red throughout the text. Thank you. Of note: the co-first author is an English speaker fluent in academic writing and has been published elsewhere. If these changes are still not suitable, we are unsure exactly of what this reviewer is requesting given the vague feedback. Please specify exactly what is not up to publication standards in this journal so we can make the appropriate adjustments. Thank you!
