# Supplementary material for: Acute effect of low-load resistance exercise with blood flow restriction on oxidative stress biomarkers: A systematic review and meta-analysis
Source: PLoS One. 2023 Apr 21;18(4):e0283237. doi: 10.1371/journal.pone.0283237 (PMC10121002; doi:10.1371/journal.pone.0283237)
Supplement: Attachment — Submitted filename: EditorReply.docx [file pone.0283237.s008.docx]

All comments made by reviewers were addressed and the paper can be accepted. However, I found that flow chart is not following PRISMA 2020 guidelines. In additon, authors used "Data management and study selection process" plus ""Data Extraction" which are not present in PRISMA 2020. Please revise those issues and resubmit. I hope in the next stage, I can accept your work.

Authors Reply: Editor – we appreciate your attention to detail and apologize for our formatting and labeling issues. We have addressed the issues you have brought up in our manuscript and we hope that the paper is now suitable for formal acceptance. We relabeled the sections “Selection process” (ln 168) and “Data collection process” (ln 179). We have also reformatted Figure 1 as per PRISMA 2020 guidelines. Last, we made sure to address the “de” in front of Lima in 2 of our tables. Thank you again for the opportunity to publish in your journal.
